# Laboratory Medicine 2014

**DOI:** 10.1155/2014/342418

**Published:** 2014-08-11

**Authors:** Mina Hur, Patrizia Cardelli, Giulio Mengozzi

**Affiliations:** ^1^Department of Laboratory Medicine, School of Medicine, Konkuk University, 120-1 Neungdong-ro, Hwayang-dong, Gwangjin-gu, Seoul 143-729, Republic of Korea; ^2^Department of Clinical and Molecular Medicine, Sant'Andrea Hospital, School of Medicine and Psychology, Sapienza University, Via di Grottarossa 1035/1039, 00189 Rome, Italy; ^3^Department of Laboratory Medicine, Clinical Biochemistry Laboratory, “City of Health and Science” University Hospital, Corso Bramante 88, 10126 Turin, Italy

Clinical laboratory tests are the scientific basis for the diagnosis and management of diseases. In addition to the traditional areas of laboratory testing, including clinical chemistry, hematology, microbiology, immunology, and transfusion medicine, genetic testing is also broadening its role in the clinical laboratory field. While many new research procedures and analytical methods are becoming available, they all should be strictly validated before being adopted in the clinical practice as routine assays. Clinical laboratories have an important role to play in this translational process from bench to bedside.

Based on the success of the inaugural issue in 2013, Biomed Research International annualized the special issue on laboratory medicine, and this special issue is the second one succeeding the first success. This issue includes a wide variety of laboratory-related topics as illustrated by four review articles and 15 research papers. A review article by J.-L. Choi et al. provides a comprehensive review on progresses in clinical application of platelet function tests. I. Mozos describes laboratory markers of ventricular arrhythmia risk in renal failure. Another review by T.-K. Er et al. deals with current approaches for predicting a lack of response to anti-EGFR therapy in* K-ras* wild-type patients. Y. Liu and G. Shi review the recent advances regarding the role of chemotactic G protein-coupled receptors in control of migration of subsets of dendritic cells.

Two interesting papers cover the basic field of laboratory medicine. The paper by H. Jin et al. was the first to compare the characteristics of erythrocytes derived from cord blood and granulocyte colony-stimulating factor-mobilized adult peripheral blood. H.-Y. Kim et al. showed that increased mitochondrial DNA copy number might be a useful biomarker associated with polycyclic aromatic hydrocarbons toxicity and hematotoxicity.

Seven papers deal with the growing area of molecular diagnostic applications. The paper by S. H. Kim et al. identifies* Candida guilliermondii *and* Candida famata *correctly by using matrix-assisted laser desorption/ionization-time of flight mass spectrometry and gene sequencing. Y. J. Hong et al. evaluated a multiplex real-time PCR and melting curve analysis for the detection of herpes simplex and varicella-zoster virus in clinical specimens. Y. Kim et al. evaluated three automated nucleic acid extraction systems for identification of respiratory viruses in clinical specimens by multiplex real-time PCR. An interesting paper by S. Kim et al. showed differences in disease risk estimations among three commercial genetic-testing services, implying that the genetic services need further evaluation and standardization. Another paper by S. Kim et al. analyzed* in vivo* expressions of the pharmacodynamic marker inosine monophosphate dehydrogenase (IMPDH) mRNA to investigate its usefulness in assessing effects of mycophenolic acid. Y. J. Hong et al. reported the significance of Lewis phenotyping in various tissues and concluded that the gastric Lewis phenotype must be used for the study on the association between the Lewis phenotype and* Helicobacter pylori*. C.-W. Park et al. proposed that the degree of hepatitis C virus (HCV) quasispecies measured by ultradeep pyrosequencing might be useful to predict progression of hepatocellular carcinoma in the patients with chronic HCV.

Six papers come from the conventional areas of hematology and chemistry. L. A. S. Nunes et al. established reference intervals for the hemogram and iron status biomarkers in a physically active male population. T.-D. Jeong et al. investigated the association between the reduction in the estimated glomerular filtration rate and the prevalence of monoclonal gammopathy of undetermined significance in healthy Korean males. Another paper by T.-D. Jeong et al. indicated that total cholesterol concentration is correlated with the levels of bone turnover markers, suggesting that it might predict osteoporosis in premenopausal women. T. Ruskovska et al. emphasized that the variability of results of total (anti)oxidants which are obtained using different assays should be taken into account when interpreting data from clinical studies of oxidative stress, especially in complex pathologies such as chronic hemodialysis. E. Gruszewska et al. concluded that the changes in concentrations of total sialic acid and free sialic acid during the same liver diseases indicate significant disturbances in sialylation of serum glycoproteins. Lastly, B. De Berardinis et al., on behalf of GREAT international, reported the usefulness of combining Galectin-3 and bioimpedance vector analysis in predicting short and long term events in patients admitted for acute heart failure.

Given that laboratory medicine plays a vital role in translating research findings into clinical practice, we hope that this special issue would broaden the readership of Biomed Research International and contribute to the scientific development in this field.

*Mina  Hur* *Patrizia  Cardelli* *Giulio  Mengozzi*

